# Local Peritoneal Cytokine Response IL-1β, IL-6, TNF-α in a Standardized Neonatal Rat Model of Necrotizing Enterocolitis

**DOI:** 10.3390/ijms27020658

**Published:** 2026-01-09

**Authors:** Tomasz Ciesielski, Marek Wolski, Łukasz Fus, Agnieszka Cudnoch-Jędrzejewska

**Affiliations:** 1 Laboratory of Centre for Preclinical Research, Chair and Department of Experimental and Clinical Physiology, Medical University of Warsaw, Banacha 1B, 02-097 Warsaw, Poland; agnieszka.cudnoch-jedrzejewska@wum.edu.pl; 2 Department of Pediatric Surgery, Medical University of Warsaw, Zwirki i Wigury 63a, 02-091 Warsaw, Poland; marek.wolski@wum.edu.pl; 3 Department of Pathology, Medical University of Warsaw, Pawinskiego 7, 02-106 Warsaw, Poland; lukaszpiotrfus@gmail.com

**Keywords:** necrotizing enterocolitis, neonatal rat model, interleukin-1β, tumor necrosis factor-α, interleukin-6, peritoneal fluid, cytokine profile

## Abstract

Necrotizing enterocolitis (NEC) is a life-threatening inflammatory disease of preterm infants, increasingly viewed as a cytokine-driven disorder of the immature intestine. We aimed to characterize local peritoneal concentrations of interleukin (IL)-1β, IL-6 and tumor necrosis factor-α (TNF-α) in a standardized neonatal rat NEC model and relate them to histopathological injury. Seventy-four SPRD/Mol/Lodz rat pups were allocated to a control group (CTRL; *n* = 12) or subjected to a hypoxia-hypothermia-formula-feeding NEC protocol (NEC; *n* = 62). After 72 h, small-intestinal samples were scored using a four-tier NEC scale (0–3), and peritoneal fluid cytokine levels were measured by ELISA. All CTRL animals exhibited normal histology (grade 0), whereas NEC pups showed a wide spectrum of lesions, with 66.6% classified as grade 2–3 and a significantly higher mean NEC score in NEC than CTRL (*p* < 0.001). Peritoneal IL-1β and TNF-α concentrations were markedly elevated in NEC versus CTRL animals (both *p* < 0.001), while IL-6 levels showed no statistically significant between-group difference. These findings indicate that experimental NEC in this model is accompanied by a pronounced local pro-inflammatory response dominated by IL-1β and TNF-α, whereas IL-6 may follow distinct temporal or compartment-specific kinetics. Peritoneal cytokine profiling may help refine mechanistic understanding and guide future biomarker and immunomodulatory strategies in NEC.

## 1. Introduction

### 1.1. Necrotizing Enterocolitis (NEC)

NEC forms one of the most severe gastrointestinal emergencies of the neonatal phase and is particularly characterized by very low birth weight and extremely preterm infants. In the end, NEC is a dangerous gastrointestinal crisis that primarily affects newborns with very low birth weight [1]. It is characterized by variable degrees of intestinal inflammation, coagulative necrosis and in worse cases, perforation, sepsis, multi-organ failure [2]. Despite improving neonatal intensive care, NEC is still associated with a high case-fatality rate and accounts for a significant proportion of sustained neurodevelopmental impairment and short bowel syndrome in survivors [3,4]. Population-based information indicates that NEC is present in an incidence of 5–10% of very low birthweight infants with severe geographical variation in incidence and outcomes related to population risk, clinical practice, and resource distribution [5]. The pathogenesis of NEC is multifactorial and poorly understood. Contemporary theory highlights the co-occurrence of prematurity-associated intestinal immaturity, modified microbial colonization, enteral formula feeding and ischemic–hypoxic insults to a stressed gut. Due to the immature epithelial barrier function and immature mucosal immunity in preterm infants, the intestine is the target of exaggerated innate immune responses to luminal microbes [6]. Key to this has been the mechanisms of Toll-like receptor 4 (TLR4) signaling through intestinal epithelial and immune cells: hyperactivation of TLR4 by bacterial lipopolysaccharide may cause epithelial apoptosis, lead to impaired mucosal restitution, decrease microvascular perfusion, and promote bacterial translocation [7]. At the same time, dysbiosis and a reduction in microorganisms, caused by the over-detection of potentially pathogenic taxa, promote the activation of inflammatory pathways, leading to the development of a self-reinforcing cycle of barrier damage and inflammation [8]. Experimental rodent models of NEC, typically containing hypoxia, hypothermia, and formula feeding, replicate a number of these features and have helped in identifying different molecular pathways that initiate disease and progression [9,10,11].

### 1.2. Cytokine Profile in NEC

In this pathogenic landscape, cytokines have been shown to be key mediators of the connection between microbial and environmental cues of intestinal injury. Several pro-inflammatory cytokines, including interleukin-1β (IL-1β), interleukin-6 (IL-6), tumor necrosis factor-α (TNF-α), and chemokines, including interleukin-8 (IL-8), were consistently observed to be increased in the circulation and intestinal tissues of infants with NEC and relevant animal studies. These mediators orchestrate leukocyte recruitment, strengthen local inflammation and affect epithelial and endothelial function [8,12,13,14]. Similar alterations in anti-inflammatory cytokines (IL–10 and TGF–β) and recently characterized molecules (e.g., IL-37) likewise influence the balance of injury-pro-inflammatory effectors, influencing the balance between injury and resolution [15,16,17,18]. At the apex of these inflammatory cascades, TNF-α and IL-1β occupy a central position. Both cytokines are quickly induced upon microbial products and tissue damage, and they activate downstream pathways such as NF-κB and MAPK in large parts of the body [19]. TNF-α can increase expression in resected intestinal segments and in the systemic circulation of neonates with NEC, and in experimental NEC models, where early upregulation occurs before visible tissue necrosis develops [20,21]. Functionally, TNF-α facilitates epithelial apoptosis, loose junction membrane fusion and microvascular injury leading directly to barrier destruction and tissue necrosis [22]. IL-1β has similar and synergistic actions; stimulating release of adhesion and chemokines, neutrophil recruitment, and local inflammatory insult [23]. IL-6, a pleiotropic cytokine with pro- and anti-inflammatory properties, is also influential in NEC pathobiology. Higher levels of IL-6 in plasma, peritoneal fluid and fecal samples of newborns with NEC have been described and have been known to be associated with worse disease and worse prognosis [13,24]. IL-6 fuels hepatic acute-phase response, modulates lymphocyte differentiation, and might also modify the progression from localized intestinal inflammation to systemic inflammatory phase response and multi-organ failure [25]. IL-6 and IL-8 have been suggested as potential biomarkers for early NEC detection or risk stratification in several cohorts, and with variation over the periods of observation, the disease stages and underlying sepsis probably vary [13]. Collectively, clinical and experimental data reinforce the view of NEC as a cytokine-driven disease such that an enhanced pro-inflammatory response within the immature intestines exceeds natural regulatory mechanisms. All of this has motivated interest in diagnosing and predicting the condition with cytokine profiles and therapeutic targets of cytokine pathways. Studies analyzing panels of pro- and anti-inflammatory cytokines in preterm neonates with NEC have shown complex and dynamic patterns, suggesting that the overall balance of cytokine families—rather than focusing on individual markers—may provide more insight [13,26,27]. In parallel preclinical studies, cytokine-modulating strategies such as IL-10, IL-22 and IL-37-based approaches have emerged as successful approaches to suppress excessive intestinal inflammation while not compromising host defenses [16,28,29]. In this scenario, detailed characterization of IL-1β, IL-6 and TNF-α profiles in standardized neonatal rat NEC models will elucidate their respective roles in NEC pathogenesis and elucidate diagnostic and therapeutic targets for this life-threatening disease.

### 1.3. Animal Models of NEC

In the last few decades, numerous in vivo models have been developed that have simulated major clinical and histopathological findings of necrotizing enterocolitis and which allow mechanistic and interventional investigations that are not feasible for human preterm infants. The experimental NEC is predominantly modeled in neonatal rats, mice and preterm piglets and each system provides an optimal compromise between biological relevance, technical feasibility and experimentally applicable possibilities [10]. Rodent models are popular for their cost-effectiveness, low generation time, and use of immunological and genetic agents, while the preterm piglet is considered as the most clinically useful large animal model as it is similar to human preterm infants in intestinal structure, digestive physiology and clinical course [30,31,32]. Neonatal NEC models in rats typically involve formula feeding, followed by periods of intermittent hypoxia and hypothermia to trigger segmental intestinal necrosis during this early postnatal period [33]. In modern versions, rat pups are separated from the dam right after birth, are given hyperosmolar formula by gavage, and are exposed once or repeatedly to 100% nitrogen and cold stress. These conditions result in epithelial sloughing, villous atrophy and transmural necrosis that closely resemble that of human NEC [34,35]. Those models have been widely demonstrated to describe contributions of innate immune activation, barrier failure and microvascular impairment to the pathogenesis of the disease, as well as to experiment with nutritional, pharmacological and cellular therapies. More recent modifications using a single combined hypoxia-hypothermia insult against a background of formula feeding have been proposed to minimize procedural stress while providing robust NEC induction, and to design protective measures (e.g., maternal milk) [9,36,37]. Rat model advantages include relatively high tissue yield, well-defined histological scoring systems, and the option to obtain an extensive amount of biological material (blood, peritoneal fluid, etc.) in order to profile cytokines in detail. Limitations include inter-litter variability, the necessity for manual gavage feeding and the application of environmental stressors that only partially recapitulate the complex clinical context of human prematurity [31,33,38,39]. Murine (mouse) models of NEC share many conceptual features with rat models, yet exploit the advantages of a fully tractable genetic system. In commonly utilized protocols, newborn mice were separated from the dam, gavage-fed formula, and exposed to intermittent hypoxia and cold stress, in some cases with the inclusion of certain bacterial elements or live bacteria for the purpose of hastening and homogenizing disease propagation [6,40]. Specialized murine models have also been proposed to tease out specific risk factors by, for instance, conflating severe anemia with red blood cell transfusion to reproduce transfusion-associated NEC, associating anemia-driven immune activation with NEC-like injury [41]. Graded NEC models have also been established in which different injurious stimulus intensity results in a gradient of intestinal lesions and provide a means of assessing intestinal inflammation and extra-intestinal outcomes (brain inflammation and microglial activation) [41,42]. Murine systems allow assays using knockout and transgenic strains, cell lineage tracing, and advanced immunophenotyping, but neonatal mice are so small that serial sampling and surgical procedure complexity are limited [38,43]. The preterm piglet is an especially potent translational model. Preterm delivery and intensive care, parenteral nutrition, and formula feeding are sufficient to induce spontaneous NEC that has characteristics similar to those of humans in terms of timing, localization and severity [38,44,45]. The gut immaturity and microbial colonization in premature pigs closely resemble those in preterm infants, and their systemic instability is also very similar [45]. This can be closely monitored through serial blood collection, imaging studies and even complex surgeries as performed in preterm. By contrast, this model allowed us to study the impact of various feeding strategies (e.g., colostrum versus formula or human milk oligosaccharides) relative to iron status (or blood transfusions), and probiotic or antibiotic regimens on NEC risk and severity [44,46,47]. Standardized histopathological scoring systems have been developed for preterm pig NEC that increase comparability and improve the detection of treatment effects with weighted and cumulative scores for NEC analysis [48]. However, the availability of dedicated surgical facilities, intensive care infrastructure and experienced personnel restricts the general adoption of preterm pig models outside of designated research centers. In addition to these central species, a variety of complementary approaches have been developed, which include “humanized” mouse models with engrafted human immune cells or microbiota, and both in vitro and ex vivo platforms including intestinal organoids and microfluidic “gut-on-a-chip” constructs [49,50,51]. While these methodologies are unable to completely recapitulate the systemic and hemodynamic aspects of NEC, they represent a valuable complement of approaches to disentangle cell-type-specific mechanisms, epithelial–microbial dynamics and candidate therapeutic pathways under the highly controlled conditions, and are consistent with 3R philosophy, as it minimizes the need for a high burden on in vivo experimentation. Collectively, the established animal models in this collection demonstrate that no single model system is adequate to represent the complexity of human NEC. Both neonatal rat and mouse models are of high throughput capacity and mechanistic detail, whereas preterm piglets offer better clinical and physiological fidelity [38]. Careful selection and clear explanation of the model characteristics, including species composition, timing and type of harmful stimuli, feeding schedules and histological scoring criteria, are thus necessary to ensure that experimental findings are interpreted accurately and their translational potential is maximized to manage NEC.

### 1.4. Purpose of the Study

This study aimed to quantify levels of IL-1β, IL-6 and TNF-α in a neonatal rat model specifically developed to assess NEC. We investigated these central pro-inflammatory cytokines to broaden insight into inflammatory processes responsible for NEC. The neonatal rat model was used due to its reliability and reproducibility, and its ability to reproduce major clinical and histopathological manifestations observed in human NEC; consequently, it provides sufficient biological material for detailed cytokine studies. The novelty of the present work lies in profiling inflammatory mediators in peritoneal fluid as a local compartment reflecting intra-abdominal inflammation, which is less consistently characterized than systemic circulation or intestinal tissue in experimental NEC.

## 2. Results

### 2.1. Histopathology

**Figure 1:**
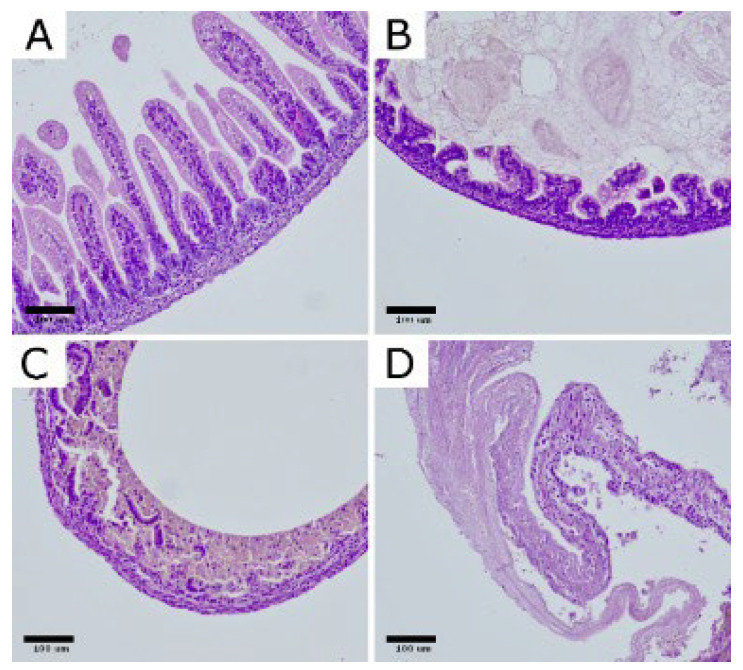
Representative histopathological changes in the intestinal wall of rats with experimental NEC. H&E-stained bowel sections illustrate the grading scale: (**A**) normal ileum (grade 0); (**B**) grade 1, partial villous atrophy; (**C**) grade 2, epithelial sloughing and/or necrosis limited to the upper portions of atrophic villi; (**D**) grade 3, complete villous loss with transmural intestinal necrosis. Images shown at 200× magnification. Scar bar: 100 μm. Photomicrographs were acquired using OPTIKA LITEView (OPTIKA; Windows x64 v2.1.24744.20240303; Ponteranica, Italy). Source: authors’ own material.

For histopathological evaluation, small intestinal specimens were collected from all animals in the CTRL (*n* = 12) and NEC (*n* = 62) groups and fixed in 10% neutral buffered formalin. Due to technical issues during tissue processing, 2 samples from the NEC group could not be analyzed, leaving a total of 12 CTRL and 60 NEC specimens for histological assessment. The preserved tissues were embedded in paraffin, sectioned at 3 µm, and stained with hematoxylin and eosin (H&E) for routine morphological assessment. NEC severity was scored using a four-tier semiquantitative grading scale: grade 0—no detectable lesions in the bowel wall; grade 1—partial villous atrophy; grade 2—epithelial sloughing and/or necrosis confined to the upper portions of atrophic villi; and grade 3—complete villous loss with transmural necrosis of the intestinal wall. (Figure 1)

In the CTRL group, all examined specimens were classified as grade 0 (12/12; 100%), confirming the absence of NEC-like changes in non-exposed pups. By contrast, samples from the NEC group showed a broad distribution of injury severity: 2/60 (3.3%) were scored as grade 0, 18/60 (30.0%) as grade 1, 14/60 (23.3%) as grade 2, and 26/60 (43.3%) as grade 3. Consequently, the intensity of histopathological damage, expressed as the mean NEC grade, was markedly higher in the NEC group than in the CTRL group (M = 2.07, SD = 0.94 vs. M = 0.00, SD = 0.00).

Given the ordinal nature of the NEC scale and the markedly skewed distribution in the CTRL group (all grade 0), between-group differences were analyzed using non-parametric methods. Using the Mann–Whitney U test, the comparison confirmed a highly significant increase in NEC severity in the NEC group relative to CTRL (*p* < 0.001). Consistently, cross-tabulation of NEC grades by group followed by a chi-square test with Fisher’s exact correction demonstrated a strong association between group allocation and NEC category (*p* < 0.001), with NEC lesions occurring exclusively in animals subjected to the NEC protocol. (Table 1).

### 2.2. Cytokine Concentrations

Peritoneal fluid concentrations of IL-1β, IL-6, and TNF-α were compared between the CTRL group (*n* = 12) and the NEC group (*n* = 62), yielding a total of N = 74 observations. Peritoneal fluid samples were available for all 62 NEC pups, including those for which histological assessment was not possible. Normality of the distributions was assessed using the Shapiro–Wilk test in the CTRL group and the Kolmogorov–Smirnov test with Lilliefors correction in the NEC group. Homogeneity of variances was evaluated with Levene’s test. Depending on whether the assumption of equal variances was met, either Student’s *t*-test for independent samples or Welch’s *t*-test was applied. The observed dispersion (SD) reflects the expected inter-individual variability of inflammatory responses in vivo within the NEC-exposed cohort.

**Figure 2:**
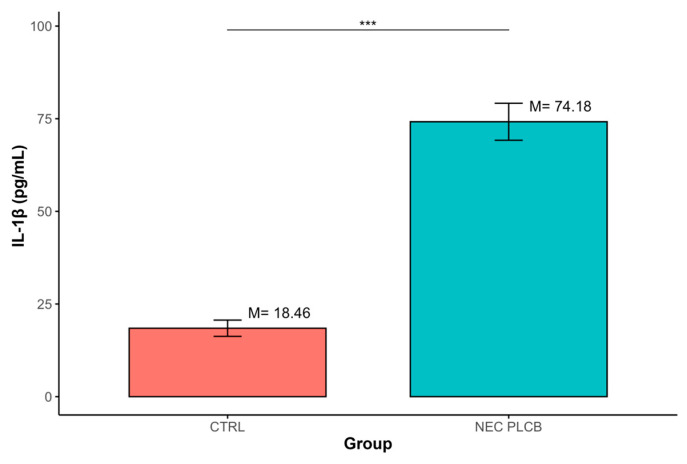
Differences in IL-1β concentrations between groups defined by the ‘Group’ variable (*** *p* < 0.001); data are presented as mean ± SD (error bars) CTRL *n* = 12, NEC *n* = 62.

The analysis demonstrated that IL-1β levels were significantly higher in the NEC group compared with CTRL (M = 74.18, SD = 39.31 vs. M = 18.46, SD = 7.59; *p* < 0.001). (Table 2, Figure 2)

**Figure 3:**
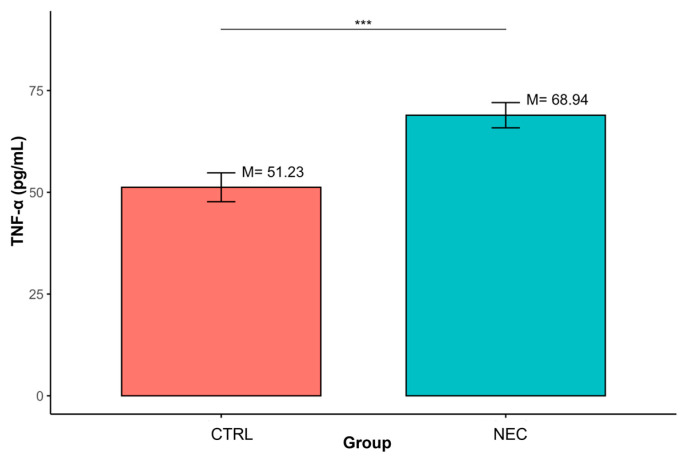
Differences in TNF-α concentrations between groups defined by the ‘Group’ variable (*** *p* < 0.001); data are presented as mean ± SD (error bars) CTRL *n* = 12, NEC *n* = 62.

Similarly, TNF-α concentrations were significantly increased in the NEC group relative to CTRL (M = 68.94, SD = 24.41 vs. M = 51.23, SD = 12.29; *p* < 0.001). (Table 3, Figure 3).

**Figure 4:**
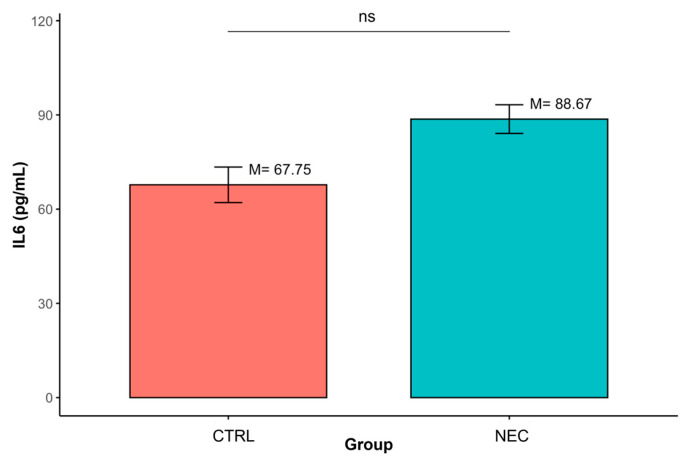
Differences in IL-6 concentrations between groups defined by the ‘Group’ variable (ns—non-significant result); data are presented as mean ± SD (error bars) CTRL *n* = 12, NEC *n* = 62.

In contrast, no statistically significant differences were observed between groups for IL-6 (M = 88.67, SD = 35.95 vs. M = 67.75, SD = 19.60; *p* > 0.05). (Table 4, Figure 4)

### 2.3. Summary of the Results

The NEC protocol demonstrated a conspicuous and measurable elevation in intestinal injury, with every CTRL animal demonstrating normal histology (grade 0), almost all NEC animals manifested NEC-like lesions and greater than two-thirds advanced lesions (grade 2–3). Accordingly, the mean NEC score was significantly greater in the NEC group compared to the CTRL group, with non-parametric tests showing a strongly significant between-group difference. As seen in this histopathological pattern, the peritoneal IL-1β and TNF-α levels were notably higher in NEC pups than in controls, while IL-6 levels were not significantly different between groups.

## 3. Discussion

In this study, we used a hypoxia–hypothermia–formula feeding model of experimental NEC in neonatal rats to characterize peritoneal cytokine profiles in relation to histopathological injury. As expected, animals subjected to the NEC protocol developed a broad spectrum of intestinal lesions, ranging from partial villous atrophy to transmural necrosis, whereas all CTRL pups remained histologically normal. On this background, we found a marked increase in peritoneal IL-1β and TNF-α concentrations in the NEC group compared with CTRL, while IL-6 levels did not differ significantly between groups. These findings support the concept that NEC in this model is driven by a robust local pro-inflammatory response dominated by IL-1β and TNF-α, and suggest that peritoneal IL-6 may have a different temporal or compartment-specific profile. By focusing on the peritoneal compartment, our results provide a standardized reference cytokine profile that may be translationally relevant to settings in which peritoneal fluid is accessible during surgical management of NEC.

NEC is increasingly viewed as a cytokine-driven disease, in which the immature intestinal mucosa of preterm infants responds excessively to microbial and ischemic–hypoxic cues, leading to uncontrolled inflammatory signaling and tissue injury. Clinical and experimental data have shown that NEC is associated with elevated levels of multiple pro-inflammatory mediators, including IL-1β, IL-6, TNF-α and various chemokines, in both systemic circulation and intestinal tissues [8,13,52]. Toll-like receptor–dependent activation of NF-κB and other downstream pathways has been identified as a key upstream event, linking dysbiosis, barrier dysfunction and epithelial apoptosis to cytokine release and progression toward necrosis and sepsis [53]. Our data, showing that virtually all pups exposed to the NEC protocol developed histological injury and that this was accompanied by robust increases in IL-1β and TNF-α in the peritoneal compartment, are consistent with this framework and further support the inflammatory nature of the disease process in this widely used rodent model.

The particularly strong signal for IL-1β and TNF-α observed in our study aligns with previous work placing these cytokines at the apex of NEC-associated inflammatory cascades. Experimental interventions that interfere with NF-κB activation or upstream TLR4 signaling have been shown to reduce intestinal expression of IL-1β, IL-6 and TNF-α and to ameliorate bowel injury in neonatal rodent models [14,54]. Similarly, neonatal rodent NEC models exposed to enteral stressors exhibit marked upregulation of intestinal IL-1β and TNF-α transcripts and protein levels, which parallel histological worsening [55]. In human studies, infants with severe NEC frequently display higher systemic IL-1β and TNF-α concentrations than controls, and the magnitude of these elevations has been associated with disease severity and adverse outcomes [13]. Functionally, both cytokines can promote epithelial apoptosis, disrupt tight junctions and impair microvascular perfusion, thereby directly contributing to barrier failure and progression from mucosal injury to transmural necrosis [12,56]. Our observation of clearly elevated peritoneal IL-1β and TNF-α in the NEC group therefore reinforces the view that these mediators are major contributors to local inflammatory damage in NEC rather than passive markers.

IL-6 did not differ significantly between groups in peritoneal fluid collected at 72 h. This finding should be interpreted in the context of compartment- and time-dependent cytokine kinetics. IL-6 may peak earlier in the course of NEC and/or may be more prominent systemically or within intestinal tissue than in the peritoneal compartment at the selected endpoint. At first sight, this seems to diverge from studies where plasma IL-6 rises early in the course of NEC and has been proposed as a candidate biomarker for diagnosis or risk stratification [24,57]. However, the IL-6 response in NEC is highly context-dependent and influenced by sampling site, timing and concomitant infectious or inflammatory events. In extremely low birth weight infants, IL-6 is often part of a broader pattern that includes IL-8, IL-10 and other mediators, and its kinetics may peak very early around the onset of symptoms or shortly before clinical deterioration [13,24,52,58]. In experimental models, IL-6 expression in intestinal tissue and serum may precede or lag behind histological injury, depending on the specific protocol and timepoints studied [59,60]. In our design, peritoneal fluid was sampled at a single timepoint 72 h after NEC induction, and it is plausible that IL-6 either peaked earlier and declined, or that its main role in this model is more systemic or tissue-bound rather than peritoneal. The lack of a statistically significant IL-6 difference should therefore not be interpreted as the absence of involvement, but rather as a reflection of temporal and compartment-specific complexity.

Our work also contributes to the ongoing appraisal of animal models of NEC. Neonatal rat and mouse models that combine formula feeding with hypoxia and hypothermia remain among the most widely used systems, offering a pragmatic balance between biological relevance, throughput and experimental flexibility [10,31,33]. These models recapitulate core histopathological features of human NEC, such as villous atrophy, epithelial sloughing and transmural necrosis, and have been instrumental in delineating the roles of innate immune activation, TLR signaling and cytokine networks [38]. The present study confirms that our modified rat protocol yields a high NEC incidence and robust inflammatory readouts, including peritoneal IL-1β and TNF-α elevation, which strengthens its utility for future interventional studies targeting specific cytokine pathways. At the same time, our findings underscore that no single model captures the full complexity of human NEC, and that results must be interpreted in light of species-specific immunology, the nature of the injurious stimuli and the timing of sampling.

Several limitations of our study should be acknowledged. First, the control group was deliberately kept small in line with 3R principles, and although the differences in IL-1β and TNF-α between CTRL and NEC were large and statistically robust, a larger CTRL sample might have allowed more precise estimation of baseline variability, particularly for IL-6. Although NEC animals exhibited a broad spectrum of histopathological injury, this study was not designed to assess cytokine levels as a function of NEC grade. Histological grading was used to validate model severity rather than to define analytical subgroups. Consequently, post hoc grade-stratified statistical comparisons were intentionally avoided to minimize the risk of data-driven inference and inflated type I error. In addition, the study was powered for group-level comparisons (CTRL vs. NEC) rather than for post hoc analyses across histopathological severity grades, which should therefore be considered exploratory. Second, a limitation of this study is that cytokines were assessed at a single predefined time point and in a single biological compartment (peritoneal fluid). NEC is characterized by dynamic, evolving cytokine signatures in blood, feces and intestinal tissue, and different mediators may peak at different stages of the disease [13,61]. Therefore, future studies incorporating longitudinal sampling (e.g., 24/48/72 h) across blood, intestinal tissue, and peritoneal fluid will be required to directly test temporal-kinetic hypotheses for IL-6 in this model. Third, the cytokine panel was predefined to focus on three key mediators (IL-1β, TNF-α, IL-6) most consistently implicated in NEC. Broader panels including anti-inflammatory cytokines (e.g., IL-10) and emerging regulatory mediators (e.g., IL-22, IL-37) may provide additional insight and should be addressed in future studies [16,29,62]. Finally, this is a single-center study in one species and one NEC protocol; while the model is well characterized, extrapolation to human NEC and to other experimental systems should be made with caution.

Despite these limitations, our study has several strengths. We used a statistically justified and histologically validated NEC model with a high incidence of disease and clearly defined grading of intestinal injury [34]. Histopathological evaluation was performed in a blinded manner using a standardized four-tier scale, and cytokine measurements were conducted with triplicate ELISA determinations. Importantly, we analyzed IL-1β, IL-6 and TNF-α in the same peritoneal samples that reflected the local inflammatory milieu in the abdominal cavity rather than relying solely on systemic measurements.

Taken together, our data highlight IL-1β and TNF-α as dominant components of the peritoneal inflammatory response in experimental NEC, while suggesting a more nuanced role for IL-6 that may depend on timing and sampling site. Future studies should integrate longitudinal cytokine profiling across blood, feces, peritoneal fluid and intestinal tissue, and expand the panel to include both pro- and anti-inflammatory mediators as well as epithelial-protective cytokines such as IL-22 and IL-37. Such work, combined with interventional strategies targeting specific cytokine pathways, may help to refine the mechanistic understanding of NEC and support the development of biomarker panels and immunomodulatory therapies for this life-threatening disease.

## 4. Materials and Methods

The study protocol received approval from the Local Ethical Committee for Animal Experiments (WAW2/093/2021). A modified rat model of necrotizing enterocolitis (NEC) involving hypoxia, hypothermia, and formula feeding was employed to induce experimental NEC and evaluate its effects on the inflammatory response. In this investigation, a total of 74 newborn SPRD/Mol/Lodz rat pups were utilized: 12 served as the control group (CTRL; no NEC protocol applied), while 62 pups were designated for the NEC group, where the complete NEC protocol was executed. Sample size calculations and underlying assumptions (endpoint, effect size, power, α) were prespecified to justify the chosen group sizes; the control group size was kept minimal in accordance with 3R principles.

The target sample size for animals subjected to the NEC protocol was established in advance based on findings from a pilot study and existing literature [34]. The primary endpoint was defined as the percentage of animals developing NEC within each group. Anticipating an incidence rate of approximately 75% among animals exposed to NEC and a reduction of about 30 percentage points in comparison groups, we calculated that a total of 60 animals undergoing the NEC protocol would be necessary when employing a chi-square test with a two-sided significance threshold of 0.05 and aiming for a statistical power of 70%. To allow for potential dropouts, 62 pups were ultimately enrolled in the NEC group.

For the non-NEC control group, an initial minimum of 10 animals was deemed adequate for exploratory comparisons against those exposed to NEC. Assuming a standard deviation equivalent to 50% of the mean, this sample size, when compared with at least 30 NEC animals, would provide roughly 70% statistical power (α = 0.05, two-sided Student’s *t*-test) to identify differences in mean values corresponding to approximately 47% of the mean or disparities in proportions around 45 percentage points (chi-square test, α = 0.05, power 70%). This was considered sufficient for addressing the exploratory endpoints outlined in the study. To mitigate potential dropouts, we aimed to recruit slightly more than the minimum required number of controls, ultimately enrolling 12 control pups. No formal criteria were set for inclusion or exclusion; allocation into groups occurred by litter, and no pups were excluded from the final analysis.

Throughout the experiment, all rats were housed in individually ventilated cages under standardized environmental conditions. The room and cage temperatures were maintained at 22 ± 2 °C with a relative humidity of 55 ± 5%, and a 12 h light/12 h dark cycle was used. The ventilation system ensured approximately 20 air changes per hour in the animal room and around 75 air exchanges per hour within each cage. Temperature and humidity were continuously monitored both in the animal room and inside the cages. Samples for subsequent analyses (including cytokine measurements) were collected after 72 h of NEC protocol duration or earlier in the case of spontaneous death.

### 4.1. NEC Protocol

The protocol was based on established formula-feeding combined with hypoxia and hypothermia paradigms, implemented in a standardized manner to achieve reproducible NEC induction while minimizing procedural stress [34]. Newborn rat pups were individually housed in ventilated cages and exposed to an atmosphere of pure nitrogen for 60 s to create temporary hypoxia, followed by a reintroduction of room air for one minute. Subsequently, the cages were transferred to a cooling chamber set at 4 °C for ten minutes to induce systemic hypothermia before returning them to their normal housing conditions. During the experimental phase, the pups received an acceptable quantity of commercially available formula milk through a 0.5 mm feeding pipette instead of maternal milk. After 72 h from the initiation of the protocol or earlier if spontaneous death occurred (not less than 24 h post-NEC induction), the pups were euthanized. Bowel samples, comprising at least three fragments from macroscopically affected portions of the duodenum, small intestine, and large intestine, were collected. The intestinal specimens were preserved in 10% neutral buffered formalin for subsequent histopathological and immunohistochemical analyses; peritoneal fluid was gathered for biochemical and cytokine evaluations.

### 4.2. ELISA Protocol for Cytokine Level Analysis

Cytokine levels of IL-1β, IL-6, and TNF-α in peritoneal fluid were measured using Quantikine ELISA kits (R&D Systems, Minneapolis, MN, USA) following the manufacturer’s instructions. Initially, each well on the microplate received 50 μL of assay diluent, followed by adding either standard solution or sample/control, also totaling to 50 μL per well. After gently mixing by tapping the plate, it was sealed and incubated at room temperature for two hours. Following this incubation period, wells were aspirated and washed five times with wash buffer while ensuring complete removal of residual liquid after each wash cycle. Next, each well was introduced to 100 μL of the appropriate cytokine conjugate (IL-1β, IL-6 or TNF-α), which was allowed to incubate again for two hours at room temperature. After another wash step where substrate solution was added (100 μL), development proceeded in darkness for half an hour before stopping with an additional solution (100 μL). Absorbance readings took place at a wavelength of 450 nm corrected against readings taken at 540 nm within half an hour post-reaction completion; triplicate analyses provided cytokine concentrations derived from standard curves specific to each analyte.

### 4.3. Histology

Tissue specimens underwent fixation using a solution of 10% neutral buffered formalin before routine processing and paraffin embedding took place. From these blocks, sections measuring three micrometers in thickness were prepared and stained with hematoxylin and eosin (H&E) for histological assessment purposes. Initial examinations utilized low power magnification (40×) to evaluate overall intestinal architecture in rats; subsequently higher magnifications (200× and 400×) allowed for more detailed investigation where lesions received semi-quantitative scoring according to a four-grade severity scale: grade zero indicating normal bowel wall without visible lesions; grade one indicating focal or partial villous atrophy; grade two indicating epithelial sloughing or necrosis limited to upper portions of damaged villi; grade three signifying total villous destruction accompanied by transmural necrosis within the intestinal wall. Histological assessment was performed by an experienced pathologist blinded to group allocation.

### 4.4. Statistical Analysis

Statistical analyses were performed using R statistical software (R Core Team, Vienna, Austria, 2023, https://www.Rproject.org/, accessed on 15 December 2024). Normality was assessed using the Shapiro–Wilk test (CTRL) and the Kolmogorov–Smirnov test with Lilliefors correction (NEC). Homogeneity of variances was evaluated with Levene’s test. Cytokine concentrations were compared between groups using Student’s *t*-test for independent samples or Welch’s *t*-test when variances were unequal. Ordinal histopathological NEC grades were compared using the Mann–Whitney U test. The distribution of NEC grades between groups was additionally evaluated using a chi-square test with Fisher’s exact correction where appropriate. All tests were two-tailed, and *p* < 0.05 was considered statistically significant.

## 5. Conclusions

In a standardized neonatal rat model of NEC based on hypoxia, hypothermia and formula feeding, we demonstrated that experimental NEC is associated with a clear increase in histopathological injury severity and a pronounced elevation of peritoneal IL-1β and TNF-α levels. Supporting the pivotal nature of these cytokines for inciting local inflammation in the abdomen, these findings are consistent with the concept of NEC as a cytokine-mediated disease of the immature intestine. In contrast, IL-6 concentrations in peritoneal fluid did not differ significantly between NEC and control animals, indicating that the role of IL-6 in this model may be more influenced by timing, compartment of measurement, or overall disease context. These findings support IL-1β and TNF-α as dominant components of the local peritoneal inflammatory response in this NEC model and provide a standardized reference profile for future biomarker and immunomodulatory studies. Further work should incorporate longitudinal, multi-compartment sampling to clarify IL-6 kinetics and translational applicability.

## Figures and Tables

**Table 1:** Histopathological grading of NEC: number of samples in each severity category.

Group	CTRL	NEC
0	12	2
1	0	18
2	0	14
3	0	26
**Total**	**12**	**60**

**Table 2:** IL-1β levels across experimental groups (pg/mL).

Group	*n*	M	SD
CTRL	12	18.46	7.59
NEC	62	74.18	39.31

**Table 3:** TNF-α levels across experimental groups (pg/mL).

Group	*n*	M	SD
CTRL	12	51.23	12.29
NEC	62	68.94	24.41

**Table 4:** IL-6 levels across experimental groups (pg/mL).

Group	*n*	M	SD
CTRL	12	67.75	19.60
NEC	62	88.67	35.95

## Data Availability

The original contributions presented in this study are included in the article. Further inquiries can be directed to the corresponding author.

## Abbreviations

The following abbreviations are used in this manuscript:

NEC	Necrotizing enterocolitis
TLR	Toll-like receptor
IL	Interleukin
TNF-α	Tumor necrosis factor-α
